# Poly(ADP-Ribose) Polymerase Inhibition in Melanoma: Biological Rationale, Emerging Clinical Evidence, and Biomarker Selection

**DOI:** 10.3390/cancers18213525

**Published:** 2026-11-03

**Authors:** Chana Peysin, Tamara A. Sussman

**Affiliations:** Department of Hematology/Oncology, Taussig Cancer Institute, Cleveland Clinic Foundation, Cleveland, OH 44195, USA

**Keywords:** melanoma, poly(ADP-ribose) polymerase inhibitors, homologous recombination, DNA damage repair, biomarkers, immunotherapy

## Abstract

Immune checkpoint inhibitors and other targeted therapies have substantially improved outcomes for patients with advanced melanoma; however, effective treatment options remain limited following treatment resistance. Poly(ADP-ribose) polymerase (PARP) inhibitors are an established therapeutic strategy in several malignancies with defects in homologous recombination repair, and emerging evidence suggests a potential role for PARP inhibition in melanoma. Alterations in homologous recombination repair and other DNA damage response pathways occur in a subset of melanomas and may create therapeutic vulnerabilities to PARP inhibition. In addition to their direct effects on DNA repair, PARP inhibitors have immunomodulatory properties that provide a biologic rationale for combination with immune checkpoint blockade. Preclinical melanoma models have demonstrated activity of PARP inhibition alone and in combination with chemotherapy, while early clinical studies have shown preliminary activity in selected patients with advanced melanoma. However, the presence of a homologous recombination repair/DNA damage response gene alteration does not necessarily indicate functional homologous recombination deficiency or predict sensitivity to PARP inhibition. This review examines the biologic rationale, preclinical and clinical evidence, and emerging combination strategies for PARP inhibition in melanoma, with particular emphasis on the challenges of biomarker selection and priorities for future clinical investigation.

## Simple Summary

Treatment for advanced melanoma has improved substantially, but many patients eventually develop resistance to available therapies. PARP inhibitors are drugs that target weaknesses in the way cancer cells repair damaged DNA and are already being used to treat several other cancers. Early studies suggest that these drugs may also benefit some patients with melanoma, but it remains unclear how to identify those most likely to respond. This review summarizes the biological rationale and current evidence for PARP inhibitors in melanoma, with particular attention to biomarkers of defective DNA repair. We discuss why the presence of a DNA repair gene mutation alone may not reliably predict treatment response and how improving these biomarkers may allow future clinical trials to better select patients who are most likely to benefit from PARP inhibitor therapy.

## 1. Introduction

Cutaneous malignant melanoma is the most lethal form of skin cancer and accounts for the majority of skin cancer-related deaths. In 2025, an estimated 104,960 new invasive melanoma diagnoses and 8430 melanoma-related deaths were projected in the United States [1]. Although early-stage disease can often be cured with surgical resection, a substantial portion of patients develop metastatic disease. Prior to the advent of immunotherapy, median overall survival (OS) for metastatic melanoma was 6–8 months, with five-year OS below 10% with conventional chemotherapy such as dacarbazine or temozolomide [2]. The development of immune checkpoint inhibitors and molecularly targeted therapies has substantially improved outcomes for patients with advanced melanoma. In the 10-year follow-up of the phase III CheckMate 067 trial, overall survival rates were 43% with nivolumab plus ipilimumab, 37% with nivolumab alone, and 19% with ipilimumab alone [3]. Similarly, combined BRAF and MEK inhibition has demonstrated durable survival benefits in patients with BRAF V600-mutant melanoma, with 5-year overall survival rates of approximately 34% in pooled analyses of the COMBI-d and COMBI-v trials [4].

Despite these therapeutic advances, treatment options remain limited for patients who develop resistance to immune checkpoint blockade and MAPK-directed therapy, highlighting the need for novel biomarker-driven strategies. Emerging genomic data suggest that a subset of melanomas harbor alterations in homologous recombination repair (HRR) and other DNA damage response (DDR) pathways. However, whether these alterations represent actionable biomarkers for poly(ADP-ribose) polymerase (PARP) inhibition remains uncertain. This review examines the biologic rationale and emerging evidence for PARP inhibition in melanoma, with particular emphasis on biomarker selection and potential combination strategies.

## 2. Molecular Classification of Melanoma

Genomic analyses classify cutaneous melanoma into four major molecular subtypes: *BRAF*-mutant, *RAS*-mutant, *NF-1* mutant, and triple wild-type [5]. These molecular alterations activate signaling pathways that promote tumor growth and have important therapeutic implications, particularly for tumors harboring actionable *BRAF* mutations.

## 3. Current Systemic Therapy for Advanced Melanoma

### 3.1. Immune Checkpoint Inhibitor Therapy

Immune checkpoint inhibitors have transformed the treatment landscape of advanced melanoma. Current immunotherapy options include anti-PD-1 monotherapy, combined nivolumab and ipilimumab, and nivolumab plus relatlimab [3,6,7,8,9].

Ipilimumab, an anti-CTLA4 antibody, was the first single agent immune checkpoint inhibitor approved for advanced melanoma in 2011 [10,11]. Subsequently, anti-programmed cell death protein 1 (PD-1) antibodies demonstrated improved efficacy and became a cornerstone of melanoma treatment. In the phase III CheckMate 067 trial, 10-year OS rates were 43% with nivolumab plus ipilimumab, 37% with nivolumab, and 19% with ipilimumab [3,12].

More recently, the phase II/III RELATIVITY-047 trial demonstrated improved outcomes with nivolumab plus the lymphocyte-activation gene 3 (LAG-3) inhibitor relatilimab compared with nivolumab alone [13,14]. Despite the durable disease control achieved with immune checkpoint blockade, effective treatment options remain limited for patients who develop resistance.

The selection of first-line systemic therapy for advanced melanoma should be individualized according to BRAF mutation status, disease burden, pace of progression, comorbidities, and patient preferences. Although immune checkpoint inhibitors are generally preferred for many patients because of their potential for durable responses, BRAF/MEK-targeted therapy remains an important option for patients with *BRAF* V600-mutant melanoma, particularly when rapid tumor regression is clinically necessary [15]. In addition, therapeutic cancer vaccines, including individualized neoantigen-based approaches, are being investigated as strategies to enhance antitumor immune responses. Early randomized clinical evidence suggests that personalized mRNA-based neoantigen vaccines combined with pembrolizumab may improve recurrence-free survival compared with pembrolizumab alone in patients with resected high-risk melanoma, although these approaches remain investigational [16].

### 3.2. Treatment After Progression

Treatment following progression on anti-PD-1 therapy depends on prior therapy, *BRAF* mutation status, patient characteristics, and disease burden. For patients who have not previously received combination checkpoint blockade, ipilimumab plus nivolumab has demonstrated greater activity than ipilimumab alone after anti-PD-1 progression with reported response rates of approximately 21–31% [17,18,19].

Targeted therapy also retains activity in the immunotherapy-refractory setting. In the German ADOREG registry, 108 patients with *BRAF* V600-mutant melanoma who progressed on first-line PD-1 therapy and received second-line BRAF/MEK inhibition achieved an ORR of 42.6%, disease control rate of 55.6%, median PFS of 6.6 months, and median OS of 16.0 months [20]. Among patients who achieve disease control with anti-PD-1 therapy and then relapse, retreatment outcomes depend on prior response; in a retrospective study (*n* = 396) response was seen in 15% of patients retreated with single-agent anti-PD-1 and 25% in those escalated to anti-PD-1 plus ipilimumab [21].

Tumor-infiltrating lymphocyte (TIL) therapy with lifileucel, FDA-approved in 2024, represents an important option for patients with good performance status who have progressed on anti-PD-1 therapy. In the phase II C-144-01 study, lifleucel demonstrated an ORR of approximately 31% with durable response in a subset of heavily pretreated patients [22].

## 4. PARP Inhibitors

### 4.1. Mechanism of Action and Synthetic Lethality

Poly ADP ribose polymerase-1 (PARP1) is a key enzyme that plays an important role in the recognition and repair of DNA single-strand breaks (SSBs), whereas BRCA1 and BRCA2 proteins are essential components of homologous recombination repair (HRR) of DNA double-strand breaks (DSBs). Loss of BRCA1 or BRCA2 function can result in homologous recombination deficiency (HRD), rendering tumor cells increasingly dependent on alternative DNA repair pathways. PARP inhibitors block repair of SSBs, leading to accumulation of DNA damage and formation of DSBs. In tumors with HRD, including *BRCA 1/2*-mutated cancers, these DSBs cannot be repaired, leading to cell death, a cytotoxic effect referred to as synthetic lethality [23]. In addition to catalytic inhibition, PARP inhibitors also trap the PARP1 and PARP2 enzymes at sites of damaged DNA, and thus prevent DNA repair, replication, and transcription [24]. PARP inhibition has demonstrated substantial clinical benefit in *BRCA*-mutated breast, ovarian, pancreatic, and prostate cancers, establishing this class as a cornerstone of targeted therapy in DNA repair-deficient malignancies.

Several PARP inhibitors have been developed for clinical use, including olaparib, niraparib, rucaparib, and talazoparib. Notably, while all PARP inhibitors demonstrate comparable catalytic inhibition of PARP1 and PARP2, their PARP trapping potency differs substantially. In descending order of PARP-trapping potency, these agents are generally ranked as talazoparib, niraparib, olaparib, and veliparib [25,26,27]. This difference in trapping potency is thought to underlie variations in cytotoxicity and clinical dosing as increased PARP trapping correlates with greater myelosuppression [28].

Olaparib was the first PARP inhibitor to enter clinical development, with the first in human trial conducted in 2009 that clinically validated the synthetic lethal interaction between PARP inhibition and BRCA1/2 deficiency [29]. Olaparib was then the first PARP inhibitor to receive FDA approval initially for *BRCA*-mutated advanced ovarian cancer [30]. Several other PARP inhibitors are now approved for clinical use, including niraparib, rucaparib, and talazoparib. These agents have established roles in selected patients with ovarian, breast, pancreatic, and prostate cancers, depending on tumor type, disease setting, and underlying *BRCA1/2* or other HRR alterations. Veliparib remains investigational and has not received FDA approval.

### 4.2. Adverse Effects of PARP Inhibitors

PARP inhibitors share common class-effect toxicities. The most common are gastrointestinal symptoms including nausea, vomiting, and diarrhea, fatigue, as well as cytopenias [31]. Anemia is the most common grade ≥3 adverse event across PARP inhibitors and niraparib is associated with the highest rate of cytopenias [31]. Rare but serious adverse events associated with PARP inhibitors include myelodysplastic syndrome and acute myeloid leukemia [32]. Overall, adverse events are generally manageable with dose modifications and supportive care [33].

### 4.3. Immunomodulatory Effects of PARP Inhibitors

Beyond their direct cytotoxic effects through synthetic lethality, PARP inhibitors possess significant immunomodulatory properties that enhance antitumor immunity and provide a rationale for combination with immune checkpoint inhibitors [34,35]. These effects happen through multiple mechanisms. One of these mechanisms is that with PARP inhibition, there is accumulation of cytosolic DNA fragments that activate the cyclic GMP-AMP synthase-stimulator of interferon gene which causes a production of interferons and chemokines including CCL5, CXCL9, CXCL10, and CXCL11 [34,35]. These chemokines recruit and activate immune effector cells within the tumor microenvironment. Preclinical studies suggest that these immunomodulatory effects may occur independently of *BRCA* mutation status [35].

An additional way that PARP inhibitors promote immunogenicity is by enhancing the tumor microenvironment by causing an increase in CD8+ T-cell infiltration and activation [36], M1 macrophage polarization, enhancing antigen presenting by dendritic cells and increasing infiltration of B cells and natural killer cells [34,37,38]. Lastly, PARP inhibitor-mediated DNA damage has been shown to increase tumor expression of PD-L1 in vitro and in vivo [39]. This observation provides additional rationale for evaluated PARP inhibitors in combination with immune checkpoint blockade.

## 5. Clinical Experience with PARP Inhibitors in Other Solid Tumors

### 5.1. PARP Inhibitors in Solid Tumors

PARP inhibitors have demonstrated clinical efficacy across multiple solid tumors, particularly those harboring DNA repair deficiencies. Clinical activity has been established in ovarian, breast, pancreatic, and prostate cancers, with *BRCA1/2* and other homologous recombination repair alterations serving as predictive biomarkers in selected disease settings. For example, in germline *BRCA1/2*-mutated advanced breast cancer, the phase III EMBRACA trial demonstrated improved PFS and ORR with talazoparib compared with standard chemotherapy [40]. Similarly, olaparib demonstrated substantial activity in metastatic castration-resistant prostate cancer (mCRPC) harboring DNA repair alterations, providing further evidence for biomarker directed PARP inhibition [41].

### 5.2. Combination Strategies: PARP Inhibitors and Immunotherapy

Given their immunomodulatory effect, PARP inhibitors have been evaluated in combination with immune checkpoint inhibitors across tumor type.

A study looking at combination niraparib (200 mg daily) and pembrolizumab (200 mg IV every 21 days) was studied in a phase 2 trial of 55 patients with advanced or metastatic TNBC. This study found an ORR of 21% and disease control rate of 49% with a tolerable safety profile [42]. Similarly, the combination talazoparib and avelumab was studied in phase 2b and 2 basket nonrandomized controlled trials in patients with advanced or metastatic solid tumors. In phase 2, the ORR was 18.2% (95% CI, 5.2–40.3%) in TNBC and 34.8% (95% CI, 16.4–57.3%) in patients with hormone receptor-positive positive, ERBB2-negative, and DNA damage repair (DDR)-positive breast cancer [43]. These findings support the potential synergy between PARP inhibition and immune checkpoint blockade.

Although these studies do not establish a synergistic effect, they demonstrate the feasibility and potential activity of combined PARP inhibition and immune checkpoint blockade and provide a rationale for further investigation of this strategy in other tumor types.

## 6. PARP Inhibitors and Melanoma

### 6.1. Preclinical Evidence in Melanoma

Although melanoma is not classically associated with *BRCA* mutations, emerging data suggest that alterations in DNA damage response and homologous recombination repair pathways may occur in a clinically relevant subset of melanomas. Preclinical studies demonstrated that PARP inhibition can induce synthetic lethality in melanoma models with deficiencies in double-strand break repair genes. For example, olaparib selectively induced apoptosis in melanoma cells with downregulation of the DSB repair gene *LIG4* and enhanced cytotoxicity when combined with dacarbazine [44]. Similarly, in the B16F10 murine syngeneic melanoma model, the PARP inhibitor veliparib, strongly potentiated temozolomide (TMZ) with significant antitumor efficacy in a dose-proportional manner, but did not show single-agent activity [45,46]. More recently, Ferretti et al. demonstrated that PARP inhibitors can restore sensitivity to MAPK inhibitors in resistant melanoma cells by reversing epithelial–mesenchymal transition like phenotype switching [47]. These findings provide a preclinical rationale for investigating PARP inhibition both as a biomarker directed strategy and in combination with BRAF/MEK inhibition in treatment-resistant melanoma.

### 6.2. Clinical Evidence of PARP Inhibition in Melanoma

Clinical data evaluating PARP inhibitors in melanoma remain limited. A randomized phase II trial evaluating veliparib with TMZ in patients with metastatic melanoma demonstrated modest improvements in PFS compared to TMZ alone (3.7 months in 20 mg of veliparib, 3.6 months in 40 mg of veliparib and 2 months in TMZ alone), although results did not reach statistical significance [48]. Exploratory biomarker analyses suggested greater activity among patients with low ERCC1 expression, wildtype *BRAF* V600 status, and positive p16 status [48]. As ERCC1 participates in the nucleotide excision repair pathway, reduced ERCC1 expression may increase susceptibility to DNA damage induced by TMZ in the setting of PARP inhibition. These data suggest that biomarker selection may influence the efficacy of PARP inhibition in combination-based therapy.

More recently, a phase II study evaluated niraparib monotherapy in 14 patients with advanced melanoma harboring alterations in homologous recombination pathway genes (*ARID1A/B*, *ARID2*, *ATM*, *ATR*, *BRCA1/2*, *BAP1* and others) who had progressed on prior anti-PD1 or BRAF/MEK inhibition. Two patients (14%) achieved a partial response and seven (50%) achieved stable disease, for a control rate of 64% [49]. Notably, both responses occurred among the 10 patients with nonuveal melanoma, whereas none of the four patients with uveal melanoma responded. The study was discontinued because of slow accrual, and its small sample size precludes definitive conclusions; however, these findings provide preliminary prospective evidence of PARP inhibitor activity in molecularly selected melanoma.

Early-phase studies of PARP inhibitor combination have shown mixed results. A phase II trial of talazoparib in combination with nivolumab conducted in seven patients with heavily pretreated/refractory disease demonstrated limited activity, although ongoing studies continue to explore this approach [50].

Additionally, a case series demonstrated partial to near-complete responses to combined PARP inhibitor and BRAF/MEK inhibitor therapy in patients with advanced *BRAF* V600E mutated melanoma that had progressed on targeted agents. These findings provide preliminary evidence of activity and suggest potential synergy between PARP and BRAF/MEK inhibition [51].

Overall, clinical evidence for PARP inhibition in melanoma remains preliminary, with modest response rates observed in early studies. In the phase II study of niraparib monotherapy, objective responses occurred in 14% of patients with HRR pathway alterations and 20% of patients with nonuveal melanoma, although these findings were based on a small cohort [49]. In contrast, the addition of veliparib to temozolomide did not significantly improve clinical outcomes compared with temozolomide alone [48]. These findings suggest that PARP inhibitors may have activity in selected patients with melanoma, but the optimal biomarkers for identifying responsive populations remain uncertain. Larger prospective studies incorporating molecular and functional measures of HRD are needed to establish the clinical benefit of PARP inhibition in melanoma.

### 6.3. Homologous Recombination Repair/DNA Damage Response Alterations and Biomarker Selection in Melanoma

Genomic analyses suggest that alterations in HRR/DDR pathways occur in a meaningful subset of melanomas, although reported prevalence varies according to the genes included and the criteria used to define pathogenic alterations. In an analysis incorporating multiple melanoma genomic datasets, Kim et al. reported that 21.4% of patients with melanoma have at least one functional HRR/DDR mutation. They most commonly involve *BRCA1*, *ARID1A*, *ATM*, *ATR*, and *FANCA*. They also found that HR-DDR gene mutation was associated with a high tumor mutational burden and responded well to checkpoint blockade [52]. Another study found that germline pathogenic/likely pathogenic variants were identified in 15.3% of individuals with melanoma with nearly half (46.9%) of these variants occurring in homologous repair deficiency genes [53]. This represents a significant proportion of patients with metastatic melanoma that could potentially benefit from PARP inhibitor therapy.

Huang et al. identified pathogenic germline mutations in all cancers using The Cancer Genome Atlas. Out of 470 skin cutaneous melanoma patients that have germline sequencing data available, 52 (11%) have pathogenic/likely pathogenic germline mutations, and 25 patients (5.3%) have mutations in *BRCA1/2* or “BRCA-ness” [54]. Looking at somatic mutations involving *BRCA1/2* or “BRCA-ness” genes in melanoma in TCGA, 16% (*n* = 72) possess actionable/likely actionable mutations when variants of undetermined significance (VUS) are excluded. If VUS are included, 58% (*n* = 255) of patients possess likely actionable mutations [55,56]. Although these findings suggest that alterations in DNA double-strand break repair genes may occur in a substantial proportion of melanomas, the prevalence of potentially actionable HRD remains uncertain, as the presence of these alterations does not necessarily predict sensitivity to PARP inhibition.

Beyond individual HRR/DDR gene alterations, genomic measures of HRD may provide additional insight into potential therapeutic vulnerabilities in melanoma. In a recent analysis of 2508 skin cancer samples, Nassief et al. evaluated genomic loss of heterozygosity (LOH) as a marker of HRD and found that LOH-high status generally did not correlate with mutations in canonical HRR genes. Notably, the prevalence of LOH-high status differed across melanoma subtypes, occurring in 30.9% of mucosal melanomas, 13.1% of acral melanomas, and 8.3% of cutaneous melanomas. These findings suggest that mutation-based testing alone may fail to identify tumors exhibiting genomic features associated with HRD, particularly in mucosal and acral melanoma. However, genomic LOH reflects accumulated genomic instability and does not necessarily establish ongoing functional HRD or predict sensitivity to PARP inhibition [57]. Further studies are needed to determine whether LOH-based assays, alone or in combination with HRR/DDR gene alterations, can reliably identify melanoma patients who may benefit from PARP inhibitor therapy.

Although these genomic studies demonstrate that HRR/DDR alterations are not uncommon in melanoma, the presence of a mutation in an HRR-associated gene does not necessarily establish functional HRD or predict sensitivity to PARP inhibition. The biologic consequences of alterations vary by gene and may depend on factors such as pathogenicity, zygosity, and loss of the wild-type allele. Therefore, defining which genomic or functional biomarkers best identify PARP-sensitive melanoma remains an important area of investigation.

Based on the available evidence, patients with melanoma harboring pathogenic alterations in key HRR genes, particularly *BRCA1/2*, may represent a potential population for further investigation of PARP inhibitor therapy [49,52]. However, the predictive significance of alterations in other DDR-associated genes, including *ATM*, *ATR*, and *ARID1A*, remains uncertain, as these alterations do not uniformly result in functional HRD [49]. Patients with elevated genomic LOH scores, particularly those with mucosal or acral melanoma, may represent an additional population of interest, although the relationship between LOH-high status and PARP inhibitor sensitivity has not been established [57]. Future clinical trials should therefore consider incorporating both genomic alterations and functional or genomic scar-based measures of HRD to improve patient selection. Such biomarker-driven approaches may help identify subsets of melanoma patients most likely to benefit from PARP inhibition while avoiding treatment of patients whose tumors harbor DDR alterations without corresponding therapeutic vulnerability.

### 6.4. Clinical Implications and Potential Patient Populations

Although molecular profiling studies suggest that a subset of patients with melanoma may harbor features associated with HRD, the proportion who could benefit clinically from PARP inhibition remains uncertain. Reported rates of LOH-high status range from 8.3% in cutaneous melanoma to 30.9% in mucosal melanoma [57]. However, these figures should not be interpreted as estimates of PARP inhibitor eligibility or treatment responsiveness, as genomic scarring does not necessarily reflect ongoing functional HRD. Furthermore, the limited clinical evidence, including the 14% ORR observed in the small phase II niraparib study, does not permit reliable estimates of the proportion of patients who would benefit [49]. Thus, while molecular profiling may identify a population for future biomarker-directed trials, the clinical utility of these biomarkers remains to be established. A proposed framework for integrating genomic alterations, genomic scar measures, and functional evidence of homologous recombination deficiency to inform future clinical investigation of PARP inhibitors in melanoma is illustrated in Figure 1.

Table 1 summarizes the completed and ongoing trials of PARP inhibitors in Melanoma.

## 7. Key Points

- PARP inhibitors are established treatments for several cancers and are now being investigated as a potential therapeutic strategy in melanoma.
- Early laboratory and clinical studies suggest that some melanomas may be sensitive to PARP inhibition, either alone or in combination with immunotherapy or targeted therapy.
- Identifying which patients are most likely to benefit remains a major challenge and will be critical to the design of future clinical trials.

## 8. Conclusions

Outcomes for patients with advanced melanoma have improved substantially with immune checkpoint inhibitors and targeted therapies; however, effective treatment options remain limited for patients who develop resistance to these therapies. PARP inhibitors have demonstrated clinical benefit in several malignancies with defects in homologous recombination repair, providing a rationale for investigating this therapeutic strategy in melanoma. Preclinical studies have demonstrated activity of PARP inhibition in melanoma models, while early clinical studies have shown preliminary evidence of activity both as monotherapy in molecularly selected patients and in combination with other systemic therapies.

Genomic studies suggest that alterations in homologous recombination repair and other DNA damage response pathways occur in a clinically relevant subset of melanomas. However, the presence of an HRR/DDR gene alteration does not necessarily indicate functional homologous recombination deficiency or predict sensitivity to PARP inhibition. Identifying biomarkers that reliably define PARP-sensitive melanoma will therefore be critical to the development of this therapeutic approach. Prospective studies are needed to define the efficacy of PARP inhibitors in appropriately selected patients and to determine the role of combination strategies, including PARP inhibition with immune checkpoint blockade or MAPK-directed therapy. At present, PARP inhibitors remain investigational in melanoma, but emerging preclinical and clinical data support continued evaluation of this strategy.

## Figures and Tables

**Figure 1:**
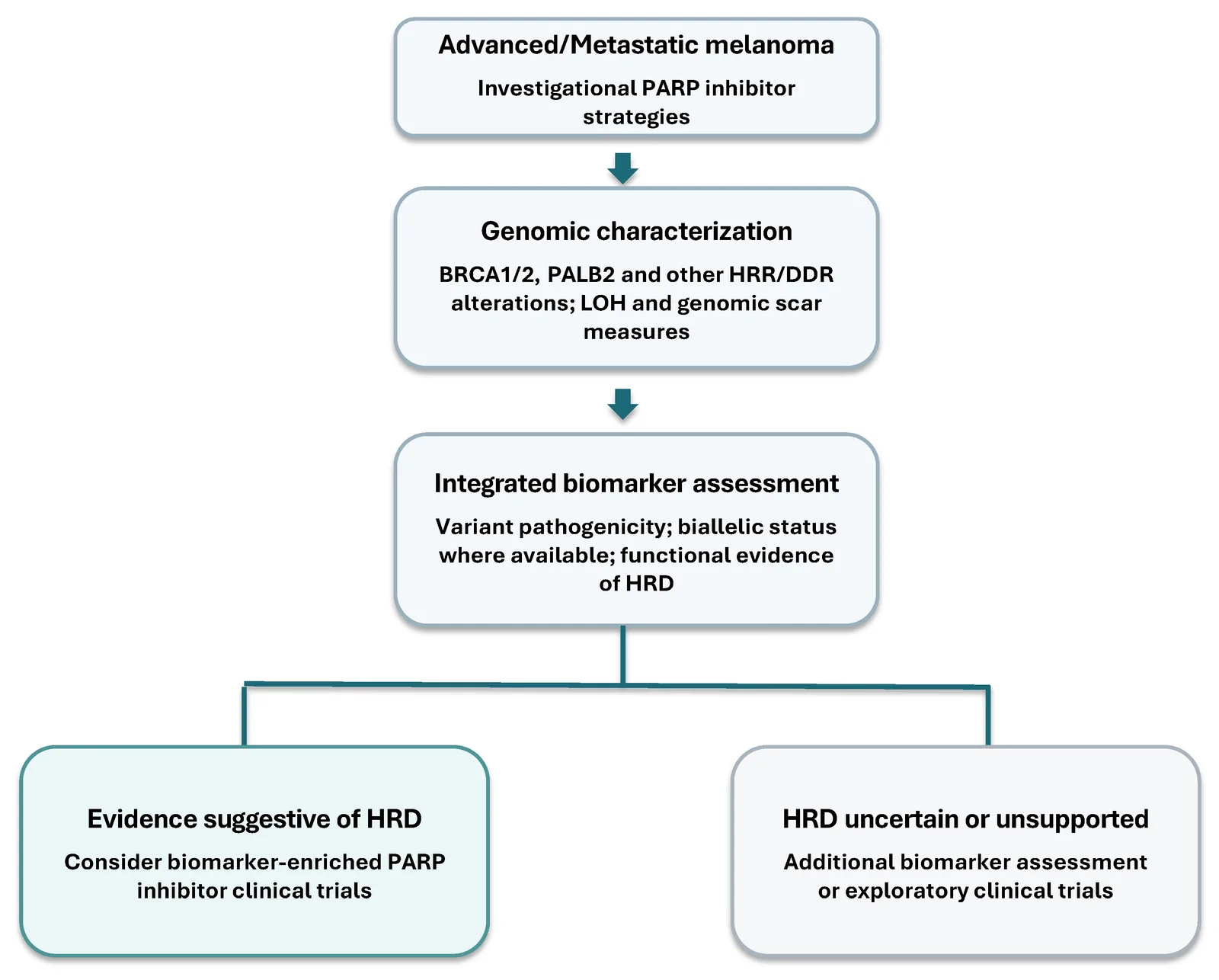
Proposed biomarker-guided framework for investigating PARP inhibitors in melanoma. This conceptual framework illustrates the integration of genomic alterations, genomic scar measures, and functional evidence of homologous recombination deficiency (HRD) to inform future clinical trial design. The framework is hypothesis-generating and has not been validated for routine treatment selection. Abbreviation key: HRD: homologous recombination deficiency; HRR: homologous recombination repair; DDR: DNA damage response; LOH: loss of heterozygosity.

**Table 1:** Published and Ongoing Clinical Studies of PARP inhibitors Relevant to Melanoma.

Trial Name/Type	Date	PARP Inhibitor	Phase	Major Results	Author, Reference
PUBLISHED TRIALS					
Veliparib + Temozolomide Phase II	2009–2010 (published in 2015)	Veliparib (ABT-888)	Phase 2	Metastatic melanoma: ORR 10.3% and 8.7% with veliparib + TMZ vs. 7.0% with TMZ alone. Median PFS 3.7 and 3.6 vs. 2.0 months; differences not statistically significant. Greater hematologic toxicity.	Middleton MR et al. [48].
Niraparib in *BAP1*/DDR-mutant tumors	2018–2021 (published in 2024)	Niraparib	Phase 2	Melanoma-enriched *BAP1*/DDR cohort: ORR 6% (1 PR); SD 44%; PD 50%. Cohort stopped at first stage.	George T et al. [58].
Niraparib in HR pathway-mutant melanoma	Accrual period not specified (published in 2025)	Niraparib	Phase 2	HR pathway-altered melanoma (*n* = 14): ORR 14% (2 PR); SD 50%; DCR 64%; median PFS 16 weeks. Nonuveal subgroup (*n* = 10): ORR 20%; no responses in uveal subgroup (*n* = 4). Small, nonrandomized study.	Kim KB et al. [49].
OLAPCO Trial (Olaparib Combinations)	Enrollment period not specified (published in 2026)	Olaparib ± ceralasertib or capivasertib	Phase 2	Mixed solid tumors with DDR alterations (*n* = 66): ORR 6.1%; clinical benefit rate 31.2%. Primary endpoint not met. These are pooled results, not melanoma-specific efficacy estimates.	Doroshow DB et al. [59].
JAVELIN PARP Medley	Enrollment period not specified (published in 2023)	Talazoparib + avelumab	Phase 2	Multicohort solid-tumor study of talazoparib plus anti-PD-L1 therapy. Melanoma-specific efficacy outcomes have not been separately reported.	Yap, TA et al. [43].
ONGOING/RECRUITING TRIALS					
NCT05524935	Active, not recruiting-results pending	Olaparib + pembrolizumab	Phase 2	Advanced uveal melanoma; efficacy results pending.	Clinical trial database
NCT02693535 (TAPUR)	Ongoing	Olaparib, talazoparib, atezolizumab + talazoparib	Phase 2	Molecularly matched, multi-tumor basket trial; melanoma-specific outcomes not reported here.	Clinical trial database
NCT05933265	Active, not recruiting	LP-184 ± olaparib	Phase 1/2	Advanced solid tumors, including melanoma; results pending.	Clinical trial database
NCT07382544	Ongoing	BMS-986504 (MRTX1719) + olaparib	Phase 1	*MTAP*-deleted solid tumors; melanoma-specific results not reported here.	Clinical trial database
NCT04693468 (TalaCom)	Ongoing	Talazoparib + palbociclib, axitinib, or crizotinib	Phase 1	Advanced solid-tumor combination study; melanoma-specific results not reported here.	Clinical trial database
NCT05327010 (ComBET)	Ongoing	Talazoparib + ZEN-3694 (BET inhibitor)	Phase 2	Advanced solid-tumor combination study; melanoma-specific results not reported here.	Clinical trial database

Abbreviation Key: BAP1: BRCA1-associated protein 1; BET: Bromodomain and Extra-Terminal motif; DDR: DNA damage repair; HR: Homologous Repair; mg: milligrams; MTAP: Methylthioadenosine phosphorylase; ORR: Objective Response Rate; OS: Overall survival; PARP: Poly(ADP-ribose) polymerase; PD: Progressive disease; PR: Partial response; PFS: Progression Free Survival; SD: Stable disease.

## Data Availability

No new data were created or analyzed in this study. Data sharing is not applicable to this article.
